# Atrophy of the brachialis muscle after a displaced clavicle fracture in an Ironman triathlete: case report

**DOI:** 10.1186/1749-7221-6-7

**Published:** 2011-10-02

**Authors:** Christoph Alexander Rüst, Beat Knechtle, Patrizia Knechtle, Thomas Rosemann

**Affiliations:** 1 Institute of General Practice and Health Services Research, University of Zurich, Pestalozzistrasse 24, CH-8091 Zurich, Switzerland; 2 Gesundheitszentrum St. Gallen, Vadianstrasse 26, CH-9000 St. Gallen, Switzerland

**Keywords:** Displaced clavicle fracture, Ironman triathlete, muscular-atrophy, brachialis muscle, brachial plexus

## Abstract

Clavicle fractures are frequent injuries in athletes and midshaft clavicle fractures in particular are well-known injuries in Ironman triathletes. In 2000, Auzou et al. described the mechanism leading to an isolated truncular paralysis of the musculocutaneous nerve after a shoulder trauma. It is well-known that nerve palsies can lead to an atrophy of the associated muscle if they persist for months or even longer. In this case report we describe a new case of an Ironman triathlete suffering from a persistent isolated atrophy of the brachialis muscle. The atrophy occurred following a displaced midshaft clavicle fracture acquiring while falling off his bike after hitting a duck during a competition.

## Background

Lesions of the brachial plexus are known to occur after displaced clavicle fractures. The most common way to get a lesion of the brachial plexus is a high-energy trauma leading to traction injuries[1,2], whereas lesions of the medial and the posterior cord have been reported most frequently[3,4]. A bone fragment from a displaced clavicle fracture is described in only 1% of the cases as the causative factor[4]. In this report we describe the case of a lesion of both, the musculocutaneous and axillar nerve with subsequent atrophy of the brachialis muscle. Regarding the anatomy, the axillar nerve originates from the posterior cord, whereas the musculocutaneous nerve originates from the lateral cord, which is not known to be affected by such injuries very often. The additional fact that a lesion of the brachial plexus occurred a certain time after a displaced midshaft fracture of the clavicle makes the case even more interesting and remarkable.

## Case presentation

**Figure 1:**
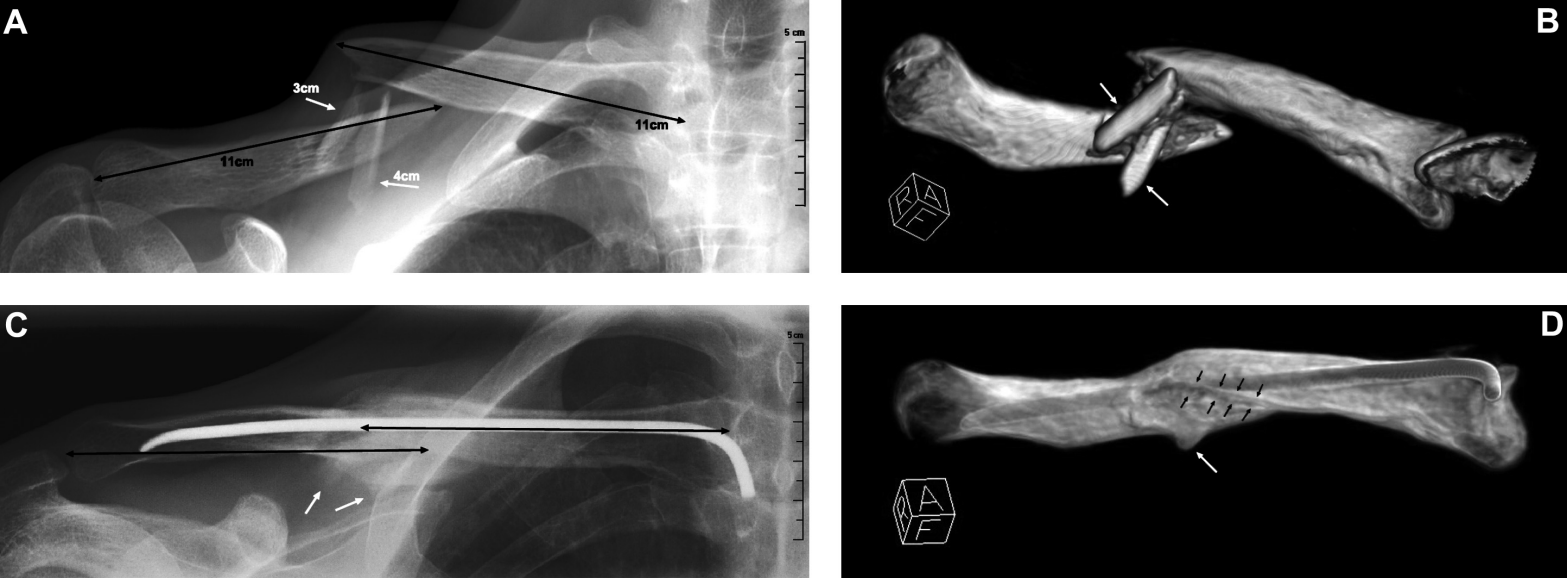
**X-Ray and 3D reconstruction of the clavicle before and after operation**. Panels A-D show pre and post operational images of the injured clavicle. A: X-ray from the displaced fracture two weeks after the accident. The black arrows indicate the two main fragments of the clavicle; the white arrows mark two additional fragments almost perpendicular to the clavicle main fragments heading towards the brachial plexus. The numbers indicate the approximate length of the fragments in cm. B: 3D reconstruction of a computer tomography done the day before the operation. The arrows indicate the fragments heading towards the brachial plexus. C: X-ray made the very first hour after the operation. The black arrows indicate the new configuration of the clavicle main fragments after open repositioning. The white arrows show the remaining fragments that could not have been removed during the operation. D: 3D reconstruction of a computer tomography done four months after the operation, before removing the nail. The black arrows show the line of consolidation and the white arrow shows the remaining residue of the fragment that could not have been removed but is almost resorbed now.

**Figure 2:**
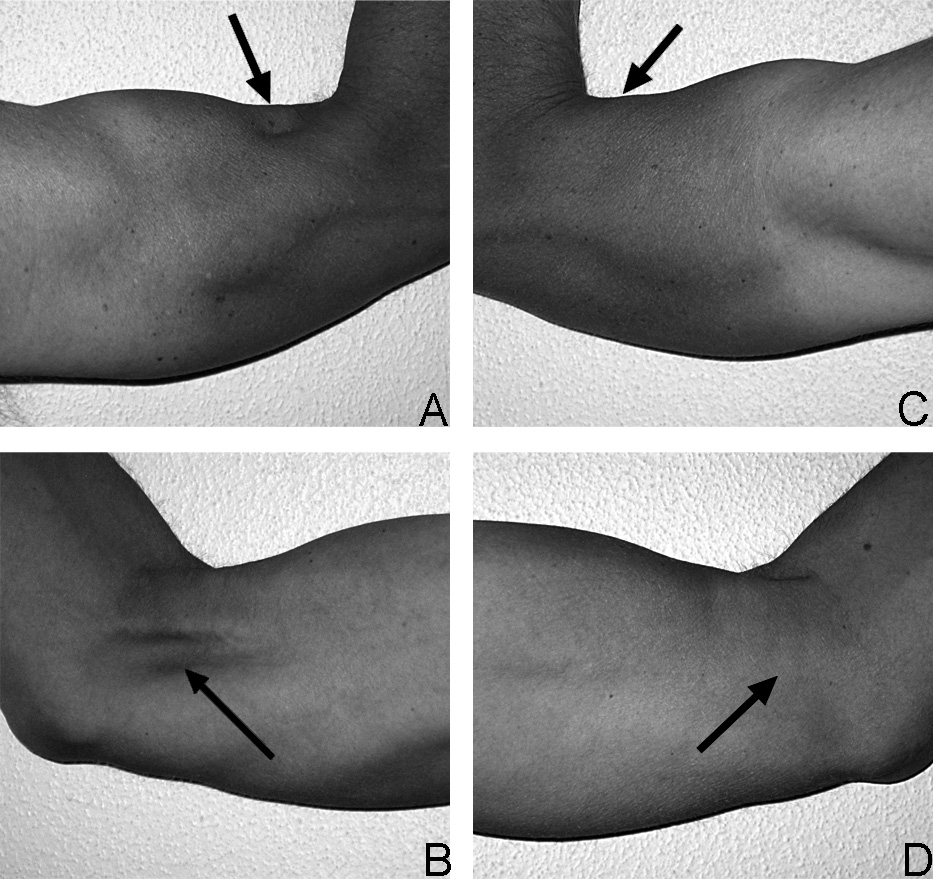
**Optical presentation of the atrophic muscle in the patient's arm**. Panels A-D show different views of the athlete's upper arms. A: lateral view of right upper arm. B: medial view of right upper arm. C: lateral view of left upper arm. D: medial view of left upper arm. Arrows in Panels A and B indicate the hole the athlete remarked on six months after operation. The topographic localization of the hole corresponds to the anatomical structure of the m. brachialis. Arrows in Panels C and D indicate the corresponding region on the healthy arm and show the normal situation without any atrophy.

In the last two kilometres of the cycling split in an Ironman triathlon a highly trained athlete hit a duck in the street and fell on his right side. He felt a sharp pain in his right shoulder and had to stop the race. Due to a previous clavicular fracture on his left side, the rider was highly suspicious of having sustained a similar injury. He returned back home and put on his old figure-of-eight dressing from the last fracture, without consulting a physician. He continued his training of indoor cycling and running and had no problems. Two weeks later before starting his swim training he continued to feel pain in his right shoulder, radiating into the radial side of the forearm and into the fingers. The clavicular head of the deltoid muscle showed a decreased sensation to light touch. An X-ray revealed a displaced fracture of the right clavicle (see Figure 1**Panel A**) and the athlete was advised to get this fracture treated surgically. A pre-operative CT scan was performed to help determine surgical fixation choices (see Figure 1**Panel B**). Since the athlete is a family physician and thus knows the available options, he asked for an intramedullary nail and the surgeon agreed. Post-surgically, the pain disappeared initially, but it returned after a few days. An MRI scan showed a small and, according to the surgeon, negligible hematoma around the plexus which was, according to the neurologist's expert opinion, the reason for the pain. Additionally, also the pressure impinging on the nerves during the accident occurrence could lead to a delayed onset of pain. Clinical and neurophysiological examination revealed a decreased sensation to light touch in the service area of the musculocutaneus nerve leading to the working hypothesis of a lesion of the musculocutaneus nerve. The neurologist's assessment showed that there were no signs of atrophy. With pregabalin - an antiepileptic drug which can be used for the treatment of neuropathic pain - (LYRICA^®^, Pfizer AG, Zurich, Switzerland), the patient was free of pain and continued training. Four months after the operation the radiological examination was repeated (see Figure 1**Panel C and D**) and the nail was removed. In the meantime the pain was gone under the treatment with pregabalin and did not reoccur after stopping the medication, thus an intraoperative exploration of the nerves or a neurolysis was waived. Two months after removing the nail, at the start of the outdoor swimming season, the athlete realized he had a hollow in his right upper arm at the place where the brachialis muscle normally is localized. Nine months after the accident the hollow was still present (see Figure 2**Panel A to D**) as well as a decreased sensation on the clavicular head of the deltoid muscle. Normal sensation returned to the radial forearm over the sensory distribution of the lateral antebrachial cutaneous nerve. However, the atrophy of the muscle remained unchanged. During all this time, the athlete suffered no decrease in muscular strength and continued training. One year after the accident, he won two long-distance triathlons in a row.

## Discussion

Nine months after the accident the athlete shows a persisting atrophy of the brachialis muscle and a decreased sensibility in the region of the clavicle part of the deltoid muscle, whereas before the operation he also felt a decreased sensibility in the radial part of the forearm. This suggests a lesion of the brachial plexus caused by the two clavicle fragments, indicated with the arrows (see Figure 1**Panel B**) involving both, the motor and sensory branches of the musculocutaneous nerve. In most cases, palsies of the brachial plexus are the result of a high-energy trauma leading to traction-injuries associated with an acute onset of the symptoms and poor prognosis, whereas the presence of a clavicle fracture in such cases is much less important[1,2]. In 1991, Della Santa described, that only 1% of brachial plexus injuries are caused by bone fragments after a clavicle fracture[4]. We assume that in this case both - the sensible as well as the motoric - parts of the musculocutaneus nerve as well as the sensible part of the axillaris nerve were hurt by the clavicle fragments, whereas the sensible part of the musculocutaneus nerve convalesced in the meantime. Therefore, this case report shows an incident with a very rare outcome. The underlying mechanism for this kind of injury was described by Auzou et al. in 2000[5] and also Rumball et al. [1] described the onset of brachial plexus palsy after a few days after a displaced clavicle fracture. Other possibilities for the appearance of the symptoms and especially for the delayed onset could be compression from hypertrophic callus[3,4] or nonunion[4,6]. Additionally, also cases of secondary brachial plexus palsies after direct compression by a bone fragment have been reported by Reichenbacher and Siebler[7]. The persistent deficiency of the motoric part of the musculocutaneous nerve explains the atrophy of the brachialis muscle the athlete observed. A hyposensitivity in the region of the deltoid muscle is the result of a lesion in the sensible part of the axillar nerve. Anatomically, the musculocutaneous nerve originates from the lateral cord of the brachial plexus and the axillar nerve from the posterior cord, respectively. Miller et al. [3] as well as Della Santa et al. [4] showed that most frequently the medial and the posterior cord of the plexus brachialis are involved in such injuries. This agrees with the involvement of the axillar nerve, but not with the involvement of the musculocutaneous nerve, leading to the conclusion, that the athlete described in this case reports suffers from an even more rarely manifestation of coincidence of clavicle fracture and plexus brachialis injury. Based on the success of the healing process the athlete has displayed so far, and also on the neurologist's expert opinion, we assume that he has a good chance of a further recovery of his neurological function. The athlete first tried his old figure-of-eight dressing as a self-treatment and later he decided on an intramedullary nailing after he was advised to have the displaced fracture being fixed. A study of the Canadian Orthopedic Trauma Society in 2007[8] showed that an operative treatment of clavicle fractures using plate fixation is better than a non-operative treatment regarding to non-union, mal-union and cosmetic aspects. Similar results have been found by Jubel et al. in 2005[9] for intramedullary nailing and Zlowodzki et al. showed in 2005[10] that conservative treatment of a displaced clavicle fracture leads to a higher number of pseudarthrosis than an operative treatment, whereas the results were independent of the surgical method. Even if Pieske et al. [11] could show in their survey-based study in 2008 that the outcome of an operative-treated displaced clavicle fracture is always better than that of a conservatively-treated one, and that intramedullary nailing should be the surgical method of choice. Debates still exist regarding which is the superior fixation method for clavicular fractures[10,12,13] and thus each individual should be evaluated independently based on its requirements and wishes. The patient should be the center of the decision-making process, and the attending doctor has to base his decision on the demands of the patient. In this case one of the athlete's most important aims was to be able to continue his training and to participate in competitions again, as soon as possible. Considering the athlete's wishes as well as his general condition and compliance, a treatment with medullary nailing was certainly indicated and was the method of choice. Along with the result that intramedullary nailing is the best available treatment for displaced clavicle fractures, Pieske et al. [11] could also show that despite of the frequency and, additionally, the large variation of clavicle fractures there is still no standardized classification on hand. According to the classification system suggested by Pieske et al. [11] the athlete suffered a type A-3 fracture, which means it was a single, midshaft clavicle fracture without any existing contact between the fracture fragments (see Additional File 1). Ultimately an X-Ray in two planes is the gold standard for rating the type as well as degree of dislocation of a midshaft clavicle fracture, and thus should always be carried out to evaluate any further steps.

## Conclusion

This case shows that a displaced fragment in a clavicle fracture can lead to a lesion of the brachial plexus with a lesion of the musculocutaneus nerve as well as the axillar nerve and subsequently to an atrophy of the brachialis muscle. Physicians should be aware of this potential complication and diagnostic imaging is a must in any type and grade of fracture to allow a diagnostic-based treatment protocol with the best possible outcome.

## Consent

Written informed consent was obtained from the patient for publication of this Case report and any accompanying images. A copy of the written consent is available for review by the Editor-in-Chief of this journal.

## Competing interests

The authors declare that they have no competing interests.

## Authors' contributions

RCA has as main author drafted the manuscript.

KB has been involved in revising the manuscript critically for important intellectual content.

KP has made substantial contributions to concept and design of the study as well as acquisition of the data.

RT has given final approval of the version to be published.

All authors have read and approved the final manuscript.

## Supplementary Material

Additional file 1 **Classification of midshaft clacivle fractures modified after Pieske**. Click here for file

## References

[B1] Rumball KM Da Silva VF Preston DN Carruthers CC Brachial-plexus injury after clavicular fracture: case report and literature review Can J Surg 1991 6 264 266 2054758

[B2] Barbier O Malghem J Delaere O Vande Berg B Rombouts JJ Injury to the brachial plexus by a fragment of bone after fracture of the clavicle J Bone Joint Surg Br 1997 6 534 536 10.1302/0301-620X.79B4.7552 9250732

[B3] Miller DS Boswick JA Lesions of the brachial plexus associated with fractures of the clavicle Clin Orthop Relat Res 1969 6 144 149 5793005

[B4] Della Santa D Narakas A Bonnard C Late lesions of the brachial plexus after fracture of the clavicle Ann Chir Main Memb Super 1991 6 531 540 10.1016/S0753-9053(05)80325-4 1725119

[B5] Auzou P Le Ber I Ozsancak C Ronziere T Magnier P Beuret-Blanquart F Hannequin D Isolated truncular paralysis of the musculocutaneous nerve of the upper limb Rev Chir Orthop Reparatrice Appar Mot 2000 6 188 192 10804417

[B6] Hansky B Murray E Minami K Körfer R Delayed brachial plexus paralysis due to subclavian pseudoaneurysm after clavicular fracture Eur J Cardiothorac Surg 1993 6 497 498 10.1016/1010-7940(93)90281-F 8217229

[B7] Reichenbacher D Siebler G Early secondary lesions of the brachial plexus--a rare complication following clavicular fracture Unfallchirurgie 1987 6 91 92 10.1007/BF02585986 3603879

[B8] Society COT Nonoperative treatment compared with plate fixation of displaced midshaft clavicular fractures. A multicenter, randomized clinical trial J Bone Joint Surg Am 2007 6 1 10 17200303 10.2106/JBJS.F.00020

[B9] Jubel A Andermahr J Prokop A Lee J Schiffer G Rehm K Treatment of mid-clavicular fractures in adults. Early results after rucksack bandage or elastic stable intramedullary nailing Unfallchirurg 2005 6 707 714 10.1007/s00113-005-0970-8 15977006

[B10] Zlowodzki M Zelle B Cole P Jeray K McKee M Group E-BOTW Treatment of acute midshaft clavicle fractures: systematic review of 2144 fractures: on behalf of the Evidence-Based Orthopaedic Trauma Working Group J Orthop Trauma 2005 6 504 507 10.1097/01.bot.0000172287.44278.ef 16056089

[B11] Pieske O Dang M Zaspel J Beyer B Löffler T Piltz S Midshaft clavicle fractures--classification and therapy. Results of a survey at German trauma departments Unfallchirurg 2008 6 387 394 10.1007/s00113-008-1430-z 18351312

[B12] Klonz A Hockertz T Reilmann H Clavicular fractures Unfallchirurg 2001 6 70 81; quiz 80 10.1007/s001130050691 11381765

[B13] Jubel A Andermahr J Faymonville C Binnebösel M Prokop A Rehm K Reconstruction of shoulder-girdle symmetry after midclavicular fractures. Stable, elastic intramedullary pinning versus rucksack bandage Chirurg 2002 6 978 981 10.1007/s00104-002-0544-z 12395155

