# Supplementary figures and images for: Atrophy of the brachialis muscle after a displaced clavicle fracture in an Ironman triathlete: case report
Source: J Brachial Plex Peripher Nerve Inj. 2011 Oct 2;6:7. doi: 10.1186/1749-7221-6-7 (PMC3201889; doi:10.1186/1749-7221-6-7)

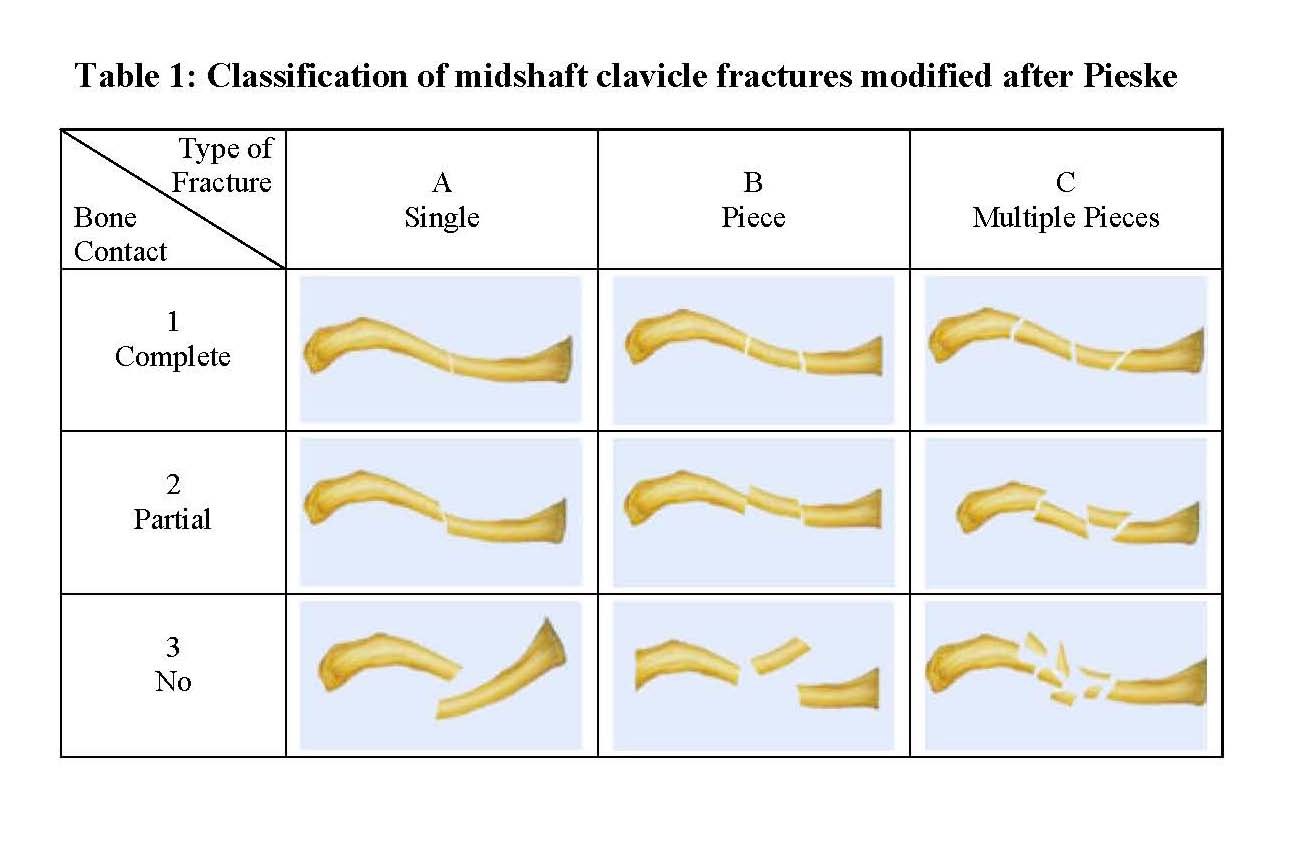

Supplement: Additional file 1 — Classification of midshaft clacivle fractures modified after Pieske. [file 1749-7221-6-7-S1.jpeg]
